# Network and Pathway-Based Integrated Analysis Identified a Novel “rs28457673–miR-15/16/195/424/497 Family–IGF1R–MAPK Signaling Pathway” Axis Associated With Post-stroke Depression

**DOI:** 10.3389/fcell.2020.622424

**Published:** 2021-01-26

**Authors:** Yan Li, Zhi-chao Wang, Ming-xi Zhu, Gui-bo Fan, Gao-shuo Xu, Tian-yang Zhao, A-yang Zhao, Shang-wei Ning, Si-hua Qi

**Affiliations:** ^1^Department of Anesthesia, The 4th Affiliated Hospital of Harbin Medical University, Harbin, China; ^2^Department of Urology, Ningbo Yinzhou No.2 Hospital, Ningbo, China; ^3^Department of Anatomy, School of Basic Medicine and Life Science, Hainan Medical University, Hainan, China; ^4^College of Bioinformatics Science and Technology, Harbin Medical University, Harbin, China

**Keywords:** post-stroke depression (PSD), pathway, network, risk gene, miRSNP

## Abstract

Single-nucleotide polymorphisms (SNPs) of microRNA (miRNA) (miRSNP) are SNPs located on miRNA genes or miRNA target sites, which have been supposed to be involved in the development of central nervous system diseases by interfering with miRNA-mediated regulatory functions. However, the association of miRSNP with post-stroke depression (PSD) has not been well-investigated. In this study, we collected 54 PSD risk genes *via* manual literature-mining and integrated PSD-related risk pathways based on multiple public databases. Furthermore, we systematically screened candidate functional miRSNPs for PSD and integrated a miRSNP-based PSD-associated pathway network, which included 99 miRNAs that target 12 PSD risk pathways. We also reviewed the association between three risk pathways and PSD pathogenetic mechanism thoroughly. Combining literature mining and network analysis, our results proposed an underlying mechanism of “miRSNP → miRNA → risk gene → pathway” axis effects on PSD pathogenesis, especially for rs28457673 (miR-15/16/195/424/497 family) → IGF1R → hsa04010 (MAPK signaling pathway). Our studies revealed a functional role in genetic modifier at the system level in the pathogenesis of PSD, which might provide further information for the miRSNP studies in PSD.

## Introduction

Globally, stroke is the second leading cause of mortality and has been identified as the third-most leading factor accounting for subsequent disability (Mboi et al., 2018). Post-stroke depression (PSD) has been noted to occur in nearly 30% of survivors of stroke and is one of the most frequent complications. PSD often leads to greater incidence and degree of disability, increased recurrence of stroke, and increased mortality, all of which pose significant challenges for clinicians tasked with treating patients (Hackett et al., 2005). Emerging experiments have demonstrated that genetic risk plays an important role in the dynamics underlying the progress of PSD. Further, many types of single-nucleotide polymorphisms (SNPs) are considered as likely role players in the dynamics and mechanistics underlying the pathogenesis of PSD (Kim et al., 2012; Zhiming Zhou et al., 2015; Liang et al., 2018), which includes SNP variants in inflammatory cytokine genes (Kim et al., 2012) and for genes in the nerve growth factor family (Liang et al., 2018). Recently, molecular biology and genetic factors have facilitated insights into PSD; however, the exact mechanisms and dynamics underlying the onset, development, and progression of PSD remain unclear.

MicroRNAs (miRNAs) are a type of single-stranded RNA composed of 18–25 nucleotides and play key roles in post-transcriptional gene expression, regulation of brain function, and disease occurrence (Bhalala et al., 2013). In recent years, research has found a close relationship between miRNAs and stroke incidence. For example, miR-124, miR-140-5p, miR-210, and other miRNAs have been identified as potentially important role players. Furthermore, previous research has also indicated that abnormally expressed miRNA may facilitate or induce the onset of and may foster the progression of PSD. For instance, results from microarray-based assays indicated the presence of 54 miRNAs and 10 miRNAs that were significantly dysregulated and were indicative of early- and late-onset PSD, respectively (Liang et al., 2019). Moreover, findings have indicated that miR-140-5p was up-regulated in PSD, and receiver operating characteristic (ROC) curves indicated that miR-140-5p predicted the occurrence of late-onset PSD with a highest sensitivity of 83.3% and a high specificity of 72.6% [Area Under the Curve (AUC) = 0.813, *P* < 0.0001]. Furthermore, overexpression of miR-140-5p in PSD also induced the inhibition of neurogenesis and capillary density. Altogether, these and other similar findings indicated that miRNAs play an important role and may significantly affect the dynamics underlying the pathogenesis of PSD.

MiRNAs are thought to mainly exert their functionality through complementarity base pair binding with the 3′ regulatory region of mRNA transcripts (Bartel, 2009). Thus, it is feasible that miRNA-associated SNPs (miRSNPs) might affect the expression of miRNA and dynamics of its processing. Based on their locales, the classification of miRSNPs into SNPs within miRNA producing genes and within miRNA target sites has been made (Saunders et al., 2007). Accumulating evidence has suggested that miRSNPs are significant role players in the pathogenic dynamics of the central nervous system (CNS), such as stroke, depression, and so forth. For instance, SNP rs3735590, located within the site of binding for miR-616 and the 3′-UTR of PON1, has been identified to induce an increase in the levels of expression of PON1 and has been significantly associated with high risk for ischemic stroke (Liu et al., 2013). Additionally, SNP rs2682818 of miR-618, is known to induce a higher level of expression of target genes that have correspondingly been associated with the recurrence of ischemic stroke (Zhang et al., 2017). Another SNP, BDNF Val66Met, by way of affecting the expression of miR-146b, induced increases in the levels of Per1 mRNA, Npas4 mRNA, and Irak1 proteins, which have been associated with greater risk for depression (Hsu et al., 2015). However, thus far, the miRSNPs potentially associated with individual genes or which could influence the dynamics of molecular pathways involved in PSD are not fully elucidated.

Herein, we sought to identify a potentially interesting set of candidate functional miRSNPs and to integrate them into a miRSNP-based PSD-associated pathway network. We expected that our findings could contribute explanations of the potential mechanisms and functional roles that miRSNPs play in PSD pathogenesis.

## Materials and Methods

### Collection of Human Genes Indicative of the Risk of PSD

We acquired data for human genes from the GeneCard databases (www.genecards.org). We next systematically searched publicly available data in PubMed (www.ncbi.nlm.nih.gov/pubmed) with a focus upon PSD-related genes and PSD-related literature published before July 1, 2019. We used the following search terms and criteria “((“Depression”[Mesh]) AND “Stroke”[Mesh]) OR by using the search term post-stroke depression OR by using post stroke depression).” In all cases, we applied the “AND” setting to filter results only for English (language). Then, we carefully read the abstract and full text and collected information for genes that had indicated a risk for PSD. We filtered results for inclusion based upon the following criteria: (i) the sum total of PSD samples exceeded five (including for samples of peripheral blood, or cerebrospinal fluid, and/or brain tissue), (ii) differences between PSD afflicted patient samples and unaffiliated control patient samples were considered significant at a *P*-value of <0.05 (for measures of the levels of mRNA or proteins), and (iii) genes documented and selected were composed of SNPs known to be significantly associated with PSD afflicted patients or similar subgroups. All genes that were selected were further assessed through typical and standardized methods, such as by the use of PCR, Western blotting, and others, and were statistically significant compared with results for controls.

### Functional Enrichment Analyses of PSD Risk-Related Genes

In order to assess the possible functions of PSD risk-related genes, we used the Gene Ontology (GO) functional enrichment analysis and Kyoto Encyclopedia of Genes and Genomes (KEGG) pathway enrichment in conjunction with the clusterProfiler package in R software. We assessed measures of statistical significance by using the Benjamini and Hochberg false discovery rate (FDR) (adjusted *P* < 0.01 was considered as the level of significance).

### miRNA and miRNA Target Gene Data

We downloaded human miRNA annotations from MirBase (http://www.mirbase.org/). We obtained human miRNA target gene pairs using 10 publicly available informatics-based tools, including from TargetScan, RNAhybrid, mirSVR, RNA22, DIANA-microT, TargetMiner, PicTar5, MirTarget2, PITA, and miRanda. We selected gene pairs for target miRNA, as well as selected those predicted at least 4 of the 10 informatics tools. Then, we applied the KEGG pathway enrichment analysis to identify pathways significantly enriched with miRNA respective of individual target genes.

### Data Analysis for miRSNPs

We used information from databases, including PolymiRTS (version 3.0), miRNASNP (version 3.0), MirSNP, and MSDD (http://www.bio-bigdata.com/msdd/), to predict miRSNPs within target sites of miRNA. We also selected miRSNPs located within miRNA genes determined from three databases [miRNASNP (version 3.0), PolymiRTS (version 3.0), and MSDD]. Concerning the above types of mentioned miRSNPs, we selected potential miRSNPs validated by experiments or that we had data that indicated they may potentially affect miRNA–mRNA interactions and required that these predictions were confirmed by at least two databases.

### Cumulative Hypergeometric Distribution

We used a cumulative hypergeometric test to analyze the crosstalk of relationships between PSD risk pathways. We calculated measures for *P*-values based upon the following equation:

$$\begin{array}{l} P=\overset{X}{\underset{i=0}{\sum }}\frac{\left(\frac{j}{i}\right)\left(\frac{m-j}{n-i}\right)}{\left(\frac{m}{n}\right)}. \end{array}$$

When we analyzed pathways for measures of crosstalk, we considered that the entire human genome had m genes, one risk pathway had j genes, that another risk pathway had N genes, and lastly, x was a numerical representation of shared genes between each of these two pathways. We analyzed measures of significance for correlations between different pathways for PSD risk and for all remaining pathways. Likewise, in order to identify miRNA targeting pathways, we considered that the entire human genome had m genes, given pathways had j genes, given miRNA had N target genes, and lastly denoted x to represent the total number of target genes confirmed as being involved in the pathway. For assessments of measures of crosstalk among PSD risk pathways or miRNA targeting pathways, we used the Benjamini and Hochberg FDR (adjusted *P* < 0.01 was considered significant).

## Results

### Manually Derived Human PSD Risk-Related Genes

We collected 54 PSD risk genes via a systematic search of published literature and through manual data mining (detailed information of risk-related genes is listed within Supplementary Table 1). Results from analyses of GO indicated that PSD risk-related genes were enriched significantly with respect to: inflammatory response, serotonin receptor-based signaling pathways, and apoptosis regulation (Supplementary Table 2), which coincided with already existent knowledge about the dynamics of the pathogenesis of PSD.

### Identifying Human PSD Risk Pathways

Results from pathway enrichment analyses based upon the PSD risk-related genes indicated that there were 22 PSD risk-related pathways (Table 1). Approximately 57.4% of the risk-related genes (31/54) were statistically significantly associated with these pathways (value of FDR <0.01), which provided an overview of PSD pathogenesis. In addition, we found that most pathways were included in categories defined within “Environmental information processing → signal transduction,” which highlighted fundamental characteristics of signal transduction PSD. Likewise, most pathways were included within categories for “Organismal Systems → aging,” which highlighted the fundamental characteristics of age and its relationship in PSD.

**Table 1:** Enriched KEGG pathway of PSD risk genes.

**KEGG pathway**	***p*.adjust**	**Pathway maps**
hsa04066: HIF-1 signaling pathway	9.13E-05	Environmental information processing (signal transduction)
hsa04010: MAPK signaling pathway	9.13E-05	Environmental information processing (signal transduction)
hsa04211: Longevity regulating pathway	0.000235603	Organismal Systems (Aging)
hsa04014: Ras signaling pathway	0.000576013	Environmental information processing (signal transduction)
hsa04152: AMPK signaling pathway	0.000795236	Environmental information processing (signal transduction)
hsa05143: African trypanosomiasis	0.000849635	Human Diseases (Infectious disease: parasitic)
hsa04151: PI3K-Akt signaling pathway	0.001008779	Environmental information processing (signal transduction)
hsa04940: Type I diabetes mellitus	0.001165778	Human diseases (Endocrine and metabolic diseases)
hsa04750: Inflammatory mediator regulation of TRP channels	0.002276445	Organismal Systems (Sensory system)
hsa04213: Longevity regulating pathway - multiple species	0.00357561	Organismal Systems (Aging)
hsa04726: Serotonergic synapse	0.00357561	Organismal Systems (Nervous system)
hsa01200: Carbon metabolism	0.00357561	Metabolism (Global and overview maps)
Hsa04920: Adipocytokine signaling pathway	0.004585583	Organismal Systems (Endocrine system)
hsa01523: Antifolate resistance	0.00558566	Human Diseases (Drug resistance: antineoplastic)
hsa04015: Rap1 signaling pathway	0.005824984	Environmental information processing (signal transduction)
hsa05418: Fluid shear stress and atherosclerosis	0.005883716	Human diseases (Cardiovascular disease)
hsa01521: EGFR tyrosine kinase inhibitor resistance	0.005883716	Human Diseases (Drug resistance: antineoplastic)
hsa04932: Non-alcoholic fatty liver disease (NAFLD)	0.006946136	Human diseases (Endocrine and metabolic diseases)
hsa04610: Complement and coagulation cascades	0.006946136	Organismal Systems (Immune system)
hsa05332: Graft-vs.-host disease	0.008810959	Human disease (Immune system)
hsa05323: Rheumatoid arthritis	0.008810959	Human disease (Immune system)
hsa05215: Prostate cancer	0.009841294	Human diseases (cancers: specific types)

**Figure 1:**
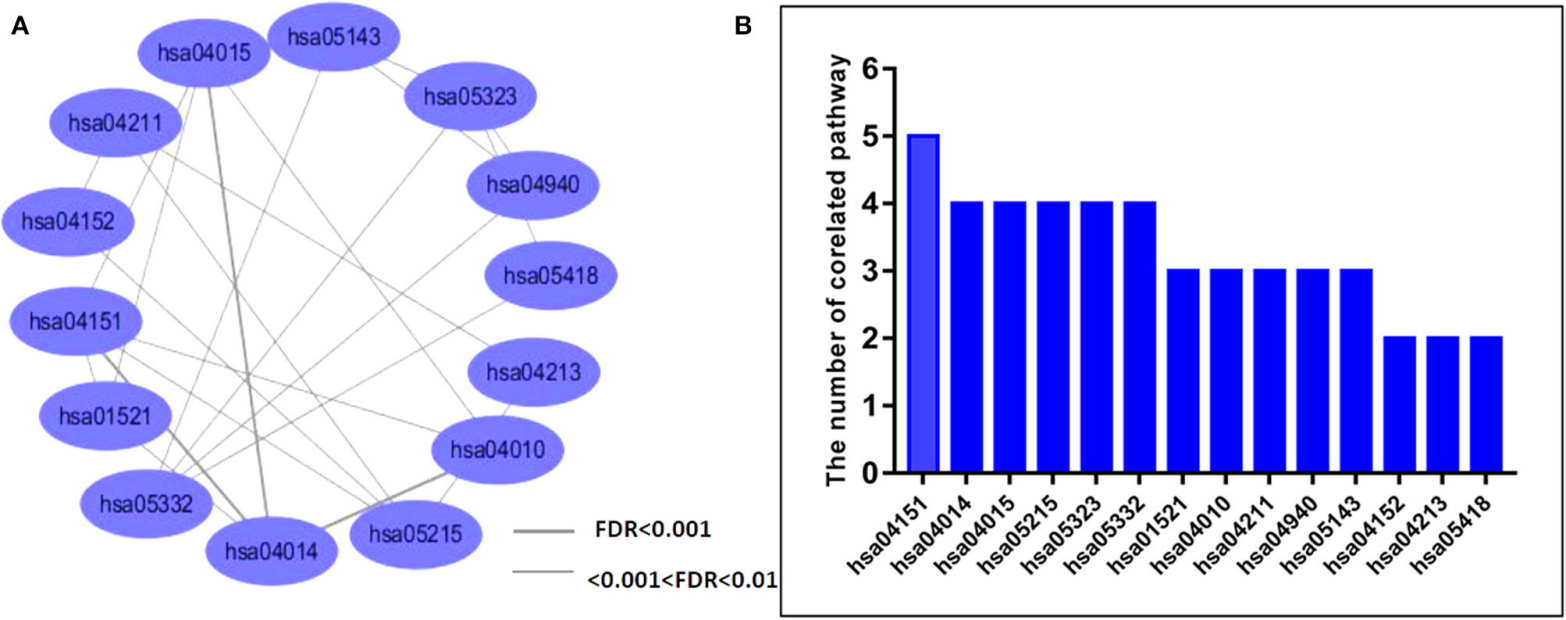
Analysis of crosstalk among PSD-related network pathways. **(A)** We analyzed crosstalk networks by cumulative hypergeometric tests and visualized results by using the Cytoscape software. Ellipses represent the pathways, lines are representative of measures of the correlation between two pathways, and the thickness of the line is representative of the strength of associations. **(B)** Bars represent the distribution of pathway crosstalk for each pathway.

We also examined measures of crosstalk, examined relationships among PSD risk-related pathways, and constructed a network of pathways that had crosstalk (Supplementary Table 3, Figure 1A). The resultant network indicated crosstalk and significant interactions for most biological pathways. There were 11 pathways that each had interactions with greater than three other pathways. Biological pathways involved in signal transduction for hsa04151 (PI3K-Akt signaling pathway) had widely interactions with five other pathways. We found that both hsa04015 (Rap1 signaling pathway) and hsa04014 (Ras signaling pathway) were themselves widely correlated with four other different pathways (Figure 1B). Collectively, our novel results indicated that the PSD risk-related pathways we identified and assessed may act synergistically and affect the dynamics of the pathogenesis of PSD, especially in regard to pathways that function in signal transduction.

### Construction of the Network for miRNA-Mediated SNP Switching Pathways

**Figure 2:**
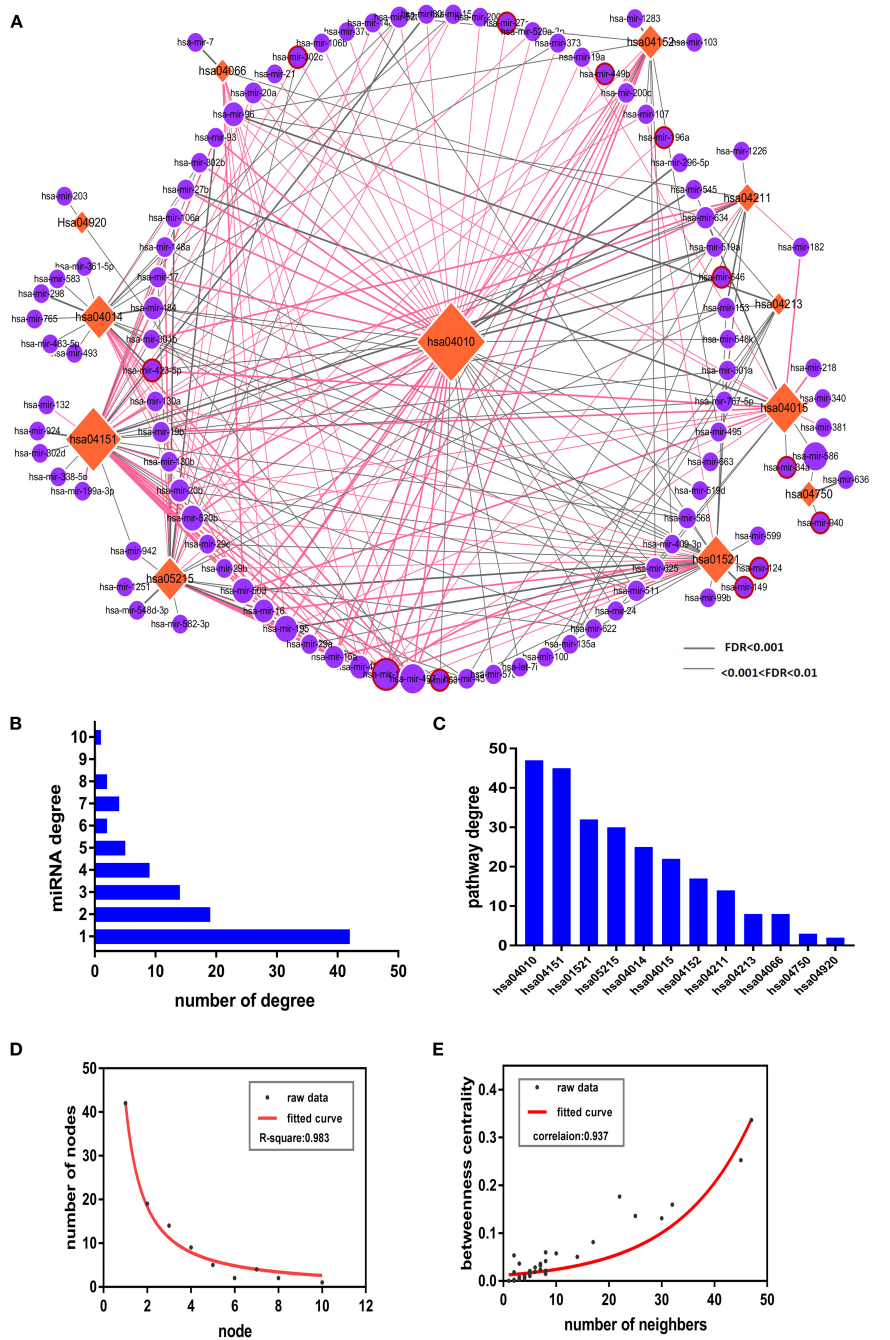
The pathway-based miRSNP switching network (PMSN) and respective topological properties. **(A)** The network of PMSN. The orange-colored rhombus is representative of the pathways, the violet circles represent the miRNA, and the differences in sizes of the node were used to represent the degree of the node. We used red lines to represent the miRSNPs within miRNA target genes for relationships between miRNAs and respective pathways containing target genes, whereas we used red circles placed around violet circles to represent the miRSNPs within miRNA genes. **(B)** The bars represent the distribution of miRNAs' degree. **(C)** The bars represent the distributions of the degrees of the pathways. **(D)** Distribution of the degrees of the nodes of PMSN and of the representative fitted curve. **(E)** Measures of distributions of betweenness centrality of PMSN and of the representative fitted curve.

An accumulating body of evidence has indicated that miRNAs play a crucial role in the dynamics underlying the pathogenesis of PSD. Thus, to better understand miRNAs' roles in PSD dynamics, we constructed a pathway network, which included 99 miRNAs, 12 PSD risk pathways, and 253 significant miRNA pathway pairs (Supplementary Table 4). Due to the possible roles of miRSNPs with respect to increasing the susceptibility and incidences of PSD, we constructed a pathway-based miRSNP switching network (PMSN), which we used to help us to assess measures of the impact of miRSNPs on PSD at a level corresponding to pathways (Figure 2A). Based upon resultant data from four relevant miRSNP databases, we retrieved candidate functional miRSNPs corresponding to 99 miRNAs. As a result, we gained an additional 42 miRSNPs located within target sites of miRNA and identified 12 miRSNPs within genes coding for miRNA and which might be consequently targeted in future research such as to assess if the targeting could affect the functions and dynamics of the pathways.

Next, we completed in-depth analyses of the PMSN. In order to first calculate the degree of risk, we assessed PSD-related pathways (Figure 2B) and miRNA (Figure 2C) and identified particular miRNAs or pathways with the highest linkages. Four pathways including for (1) hsa04010: MAPK signaling pathway, (2) hsa04151: PI3K-Akt signaling pathway, (3) hsa01521: EGFR tyrosine kinase inhibitor resistance, and (4) hsa05215: prostate cancer were in sum found to have accounted for ~79% (78 out of 99) of the total miRNAs. These results indicated that the above four pathways were increasingly susceptible to regulation by the associated miRNAs. The miRNA degree analyses (Figure 2B) indicated that 38 miRNAs interacted with greater than 3 PSD risk pathways. An interesting finding was that miR-15b regulated 10 PSD risk-related pathways (Figure 2A), which indicated that they may have potential prominent roles as genetic regulators influencing measures of the pathogenesis of PSD. Furthermore, we assessed topological properties resultant from network-based analyses of PMSN. Distribution of degrees (Figure 2D) and betweenness centrality distributions (Figure 2E) both indicated that the PMSN had characteristic features of both small-scaled networks and scale-free networks. This signifies that hub nodes, including miRNA and risk-related pathways, had higher degrees and betweenness of centrality as well as had significant roles in the dynamics of the PMSN. These results suggested that the hsa04010: MAPK signaling pathway played a potentially important role in the dynamics related to PSD as this factor had the highest measures of betweenness centrality in our network.

### Analyses of the Potential Mechanisms Underlying a Polymorphic “Switch” Influencing the Regulation of miRNAs' in PSD Risk-Related Pathways

#### Hsa04010 Pathway in PSD

**Figure 3:**
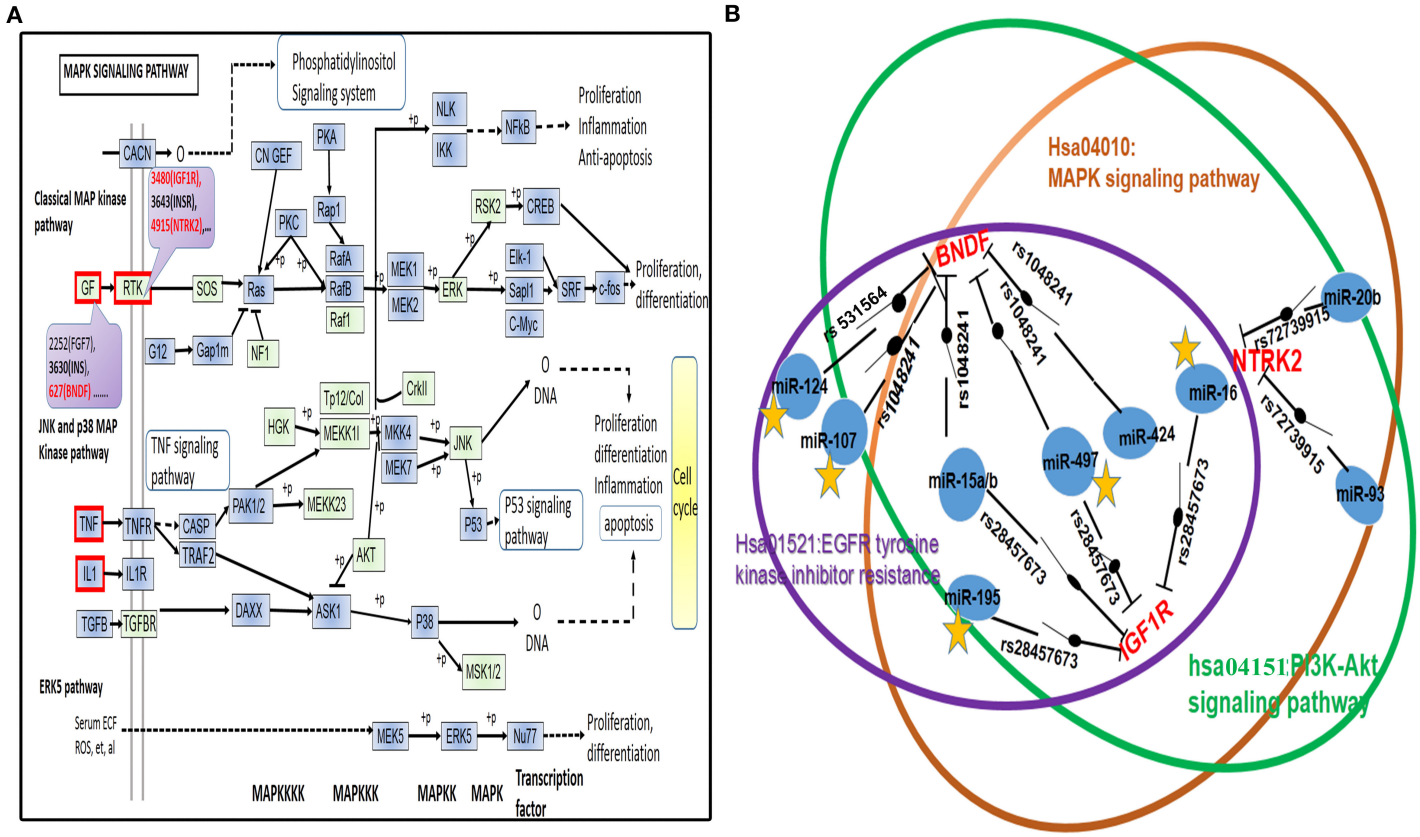
A depiction of the MAPK signaling pathway in the KEGG database. **(A)** We represented proteins or associated complexes that were encoded by PSD risk-related genes with a blue background, whereas we used red characters to represent the respective genes that encode them and that also contained miRNA target genes composed of miRSNPs located within their 3′-UTR regions. **(B)** Diagram of the schematics of the miRSNP–miRNA–gene–pathway axis. We used red lettering to represent high-risk genes and used blue circle to indicate their respectively identified regulatory miRNAs. We identified links between them seen in the connecting lines and which are indicative of the dynamics of regulatory influences of miRNAs upon the genes. The symbol representative of the switch is located upon the line showing the trend for mean values of the miRSNPs. We used the relatively large-sized circles on the peripheral in order to help denote the pathways miRSNPs that may have had important influences by way of their observed effects upon the ability of miRNAs to regulate target genes. We used orange-colored stars placed below miRNAs in order to represent the literature that reported miRNAs that have been characterized to significantly influence the regulation of target genes.

Based upon the above analyses, the hsa04010: MAPK signaling pathway had extremely important significance in the dynamics underlying the pathogenesis of PSD. As it was regulated by maximum numbers of miRNA, of which ~47% of the whole sum were found to have been predictive of miRNAs, the result highlighted its potential effects upon PMSN topological analyses. Therefore, we conducted a thorough analysis of this pathway, as well as investigated the locations of miRSNPs in the map for the KEGG pathway (Figure 3A).

#### IGF1R, BNDF, and NTRK2 in PSD

From analyses of hsa04010 (MAPK signaling pathway), we identified three PSD high-risk genes, including IGF1R, BNDF, and NTRK2 that concurrently contained miRSNPs in their respective 3′-UTR regions (Figure 3A). We further characterized measures of the potential mechanisms of the miRSNP → gene → pathway effect because the miRSNPs might impact PSD pathway by way of regulating functions of specific genes (Figure 3B). The three high-risk genes were found to be regulated by nine miRNAs, whereas five of those miRNA target gene pairs were reported through experimental validation (Figure 3B). Meanwhile, we identified seven miRSNPs within target sites for miRNA that might have affected the levels of expression of respective target genes and biological functions, which would further influence the status of hsa04010 (MAPK signaling pathway) in PSD. However, hsa04010 (MAPK signaling pathway) might not be the only signaling pathway that was influenced in such a manner, especially since the hsa04151 (PI3K-Akt signaling pathway) and the hsa01521 (EGFR tyrosine kinase inhibitor resistance) might have also been affected by IGF1R (via miR-15a, −15b, −16, −195, and −497 and their miRSNPs) and BNDF (via miR-15a, −15b, −424, and −497 and their miRSNPs). Overall, IGF1R, BNDF, and NTRK2 might have played a relatively critical role in the dynamics underlying the pathogenesis of PSD and may have been regulated by various types of miRNAs and miRSNPs, thereby consequently influencing several important PSD risk-related pathways.

### miRSNPs' Roles in the Dynamics of PSD

**Figure 4:**
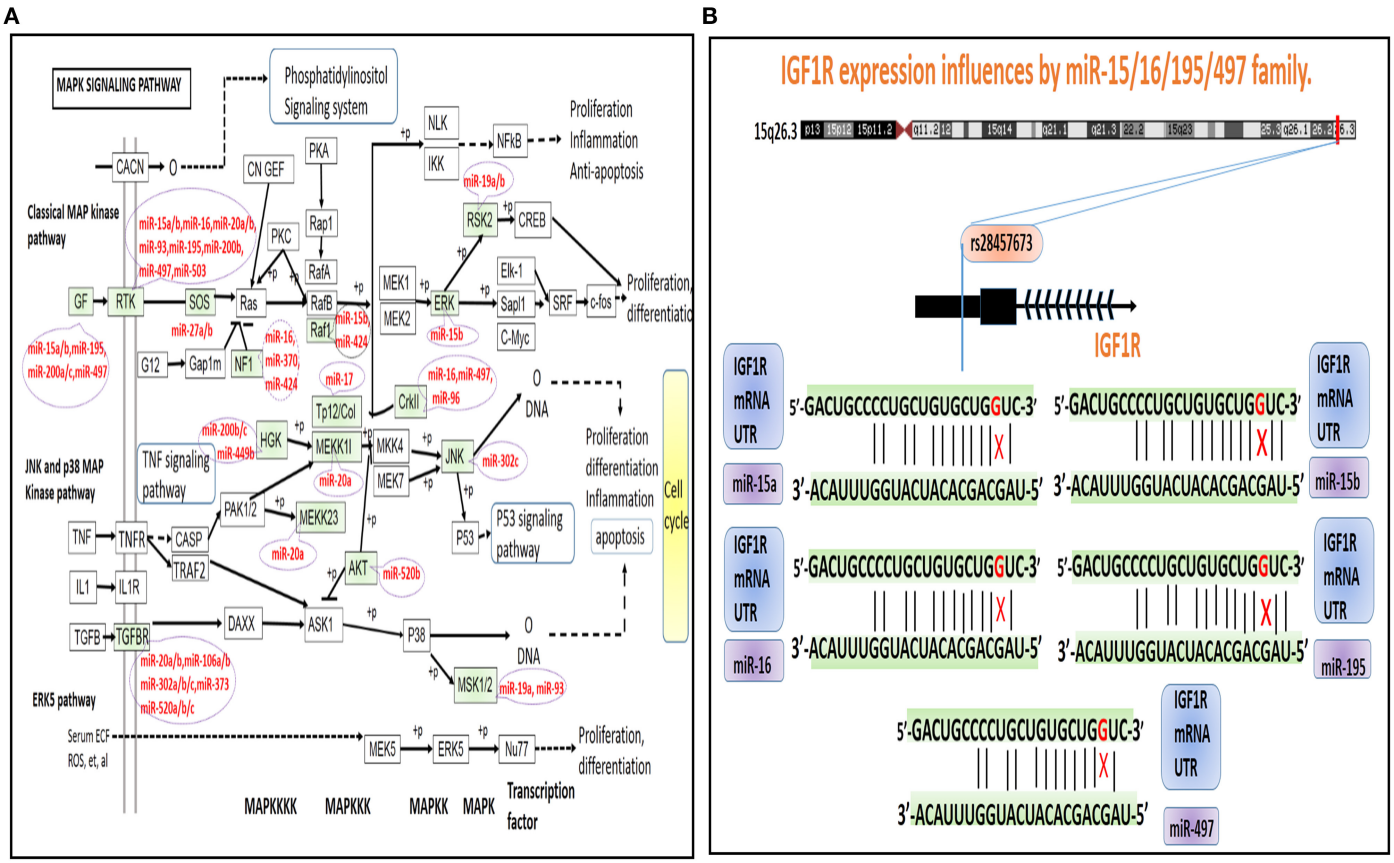
A depiction of MAPK signaling pathway dysregulation and miRNAs in the KEGG database. **(A)** The green-colored background and miRNAs are representative of miRNA target genes that are known to contain miRSNPs located within their 3′-UTR regions. **(B)** A model illustrating the potential underlying mechanisms of rs28457673 that likely played an important role in influencing the levels of expression of IGF1R via associated interactions with members of the miR-15/16/195/497 family.

Interestingly, after further in-depth analysis of these miRSNPs, we found that rs28457673 is one such variant located in the 3′-UTR of the IGF1R gene, which could affect the binding ability of IGF1R mRNA as well as affect several different miRNAs (including miR-15a/b, −16, −195, and −497) (Figure 4). We found it to be noteworthy that these miRNAs are members in a class of the family of miRNAs, including for the miR-15/16/195/424/497 family. Also noteworthy is that members of this family contain highly similar seed sequences that act so as to recognize the 3′-UTR of the target genes. Moreover, members in this family also have been reported to act in synergy such that they regulate multiple downstream target genes (Linsley et al., 2007). For instance, miR-497 is known to be able to target the 3′-UTR of IGF1R and induce the down-regulation of the levels of expression of IGF1R (Guo et al., 2013; Henghui Cheng et al., 2017); in addition, a study reported that miR-16-5p targeting IGF1R resulted in a significant induction of neutrophil apoptosis (Guo et al., 2013). Based on the above evidence, we have reason to speculate that the other three miRNAs could be able to down-regulate IGF1R. Altogether, these findings suggested that the underlying mechanism: rs28457673 (miR-15/16/195/424/497 family) → IGF1R → PSD risk-related pathways could be key role players influencing the dynamics underlying the pathogenesis of PSD.

Another important gene indicative of high-risk in CNS is BNDF, which encodes for a member of the nerve growth factor family of proteins. Meta-analyses have indicated that PSD afflicted patients had lower levels of BDNF in serum samples (Xu et al., 2018). As has been documented in the literature, several miRNAs are able to regulate the levels of expression of BDNF. Of note, miR-124 is the most understood and characterized of such types of miRNA and is known to induce the down-regulation of BDNF in human neuronal cell lines (Wang et al., 2019). Additionally, previous research has indicated that miR-124 can serve as a biomarker to diagnose depression, and that the inhibition of miR-124 may help relieve depression by way of regulating the BDNF-TrkB signaling pathway (Wang et al., 2017). rs531564 is known to be a functional SNP in MIR124-1, and known targets of miR-124 (e.g., BDNF and DRD4 genes) were able to explain measures of the effects of this miRNA upon consequent behavior (Gonzalez-Giraldo et al., 2015). Altogether, the above information enhances our understanding of the possible dynamics underlying the fundamental mechanisms and effects of the miRSNP-miR-124 → BDNF → PSD risk-related pathways.

NTRK2 is neurotrophic receptor tyrosine kinase 2; when neurotrophin binds to this receptor, it induces protein kinase-based phosphorylation including of itself and including additional members that are integral parts of the MAPK pathway. MiRSNP rs72739915 variant is within the high-risk gene TrkB (aliases for NTRK2 gene) and might affect the binding of both miR-20b and miR-93 and their target genes and then could influence the hsa04151 (PI3K-Akt signaling pathway). Research has identified miR-93 as a candidate reference miRNA gene useful for the analysis of major depressive disorder (Liu et al., 2014). Furthermore, miR-93 can regulate neurological function, edema, and apoptosis via its effects upon the TLR4/NF-κB signaling pathway based upon a study of rats with intracerebral hemorrhaging (Shang et al., 2019). Findings have also suggested that miRNA-93 can be effectively used as an indicator from blood samples to help diagnose and predict the expected functional recovery of patients at risk from and recovering from acute stroke (Ma et al., 2019). Altogether, our findings in combination with results from similarly oriented previous research help to strengthen the understanding of the underlying molecular mechanisms playing key roles and having important effects in the miRSNP-miR-20b/93 → NTRK2 → PSD risk pathways.

## Discussion

**Figure 5:**
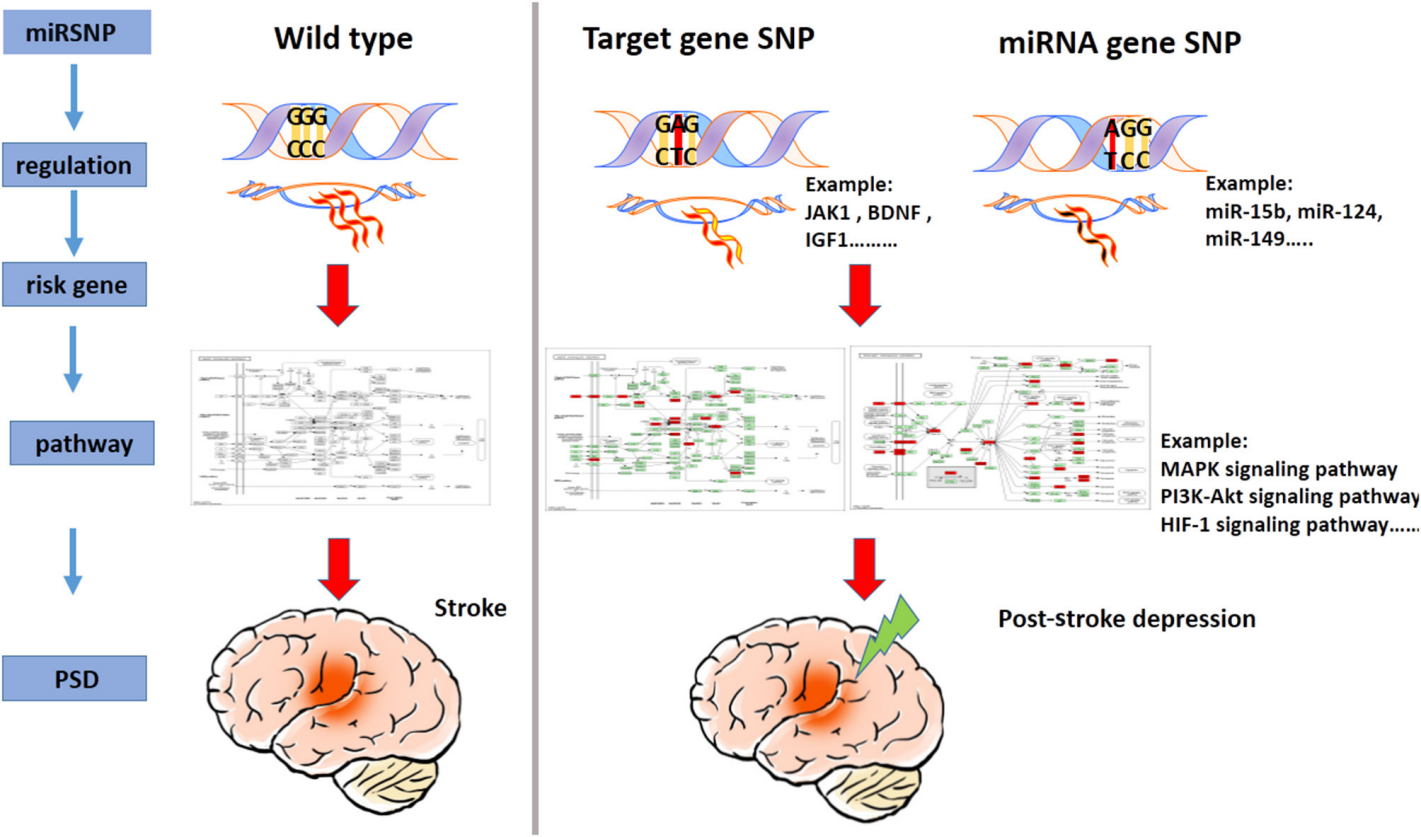
A model illustrating the miRSNP → miRNA → risk gene → pathway effect PSD.

PSD is a complex disease with a typically bad prognosis and high mortality, and the dynamics underlying its pathogenesis remain unclear. Thus, in this study, we compiled a catalog of genes related to the risk of PSD, assessed pathways that were found to be enriched in PSD, and identified miRNAs that appeared to target pathways related to PSD risk. Furthermore, by way of using miRSNP as the breakthrough point and by screening reliable miRSNP databases, our novel findings revealed the dynamics underlying the “miRSNP–miRNA–mRNA–risk pathway” axis (Figure 5). For the first time, we performed a novel systematically-based screening for potential functionally oriented miRSNPs and constructed the PMSN. These steps allowed us to elaborate upon their possible mechanisms and add to the current knowledge of PSD. To increase the depth of our study, we also analyzed the correlation with hsa04010 (MAPK signaling pathway) and PSD development, assessed measures of the significance of three genes known to be high-risk indicators for PSD, and finally examined the potentiality of mechanisms of specific miRSNPs as “switches” acting to regulate miRNAs of the PSD risk pathways.

In total, we identified 22 PSD risk pathways, including those related to the MAPK signaling pathway, serotonergic synapses, and mediators and regulators of inflammation related to TRP channels, and identified other important findings and measures, which contributed to the pathogenesis of PSD. The results may have also helped to inform latent inter-relationships between PSD and various other types of disorders, such as “hsa04610 (complement and coagulation cascades),” which was demonstrated to have had a close connection with PSD at a level corresponding to biological pathways. These findings are in agreement with reports documenting that patients with more significant measures of mean platelet volumes at the time of admission for medical care had a corresponding later onset of PSD 30 days post-stroke (Qiu et al., 2018). The clinical manifestations of PSD are diverse and heterogeneous. The novel pathway-based genetic analyses we used may help to facilitate the comprehension of how PSD evolves and progresses, may help to recognize different and even irrelevant biological processes, and may help to identify pathways responsible for pathogenesis in similar types of diseases. Our findings have broad implications and should help to facilitate the development of new target-based therapies and new individualized therapeutic approaches.

It is well-established that miRNAs facilitate the regulation of gene expression, largely at posttranscriptional levels. A single type of miRNA has the capacity to target a variety of genes, and as a result, miRNA may be involved in regulating various biological pathways, whereas a pathway can be targeted by several miRNAs (Xu et al., 2011). We were able to identify 99 miRNAs, which hold the potential for use in helping to regulate risk pathways for PSD and which we documented to have been more significant than were miRNAs targeting individual genes related to PSD risk. This is also consistent with the results for expression of circulating microRNA profiles in post-stroke patients with early onset of PSD and for target genes of these miRNAs that were found to have been enriched with respect to MAPK-related signaling pathways (Zhang et al., 2016). Accumulating evidence has also indicated that genetic variation in 3′-UTR contributes to the etiology of human diseases (Latini et al., 2017; Wigner et al., 2019; Vad et al., 2020). In recent years, miRSNPs have been recognized as potential genetic risk factors that have been rapidly identified and investigated. However, the frequency of SNPs located in miRNAs is very low. Hence, a study linking miRSNPs to disease that provides a convincing result requires a large sample size and underscores the need to detect low frequencies of variation in genetic-based research (Schizophrenia Psychiatric Genome-Wide Association Study Consortium, 2011). Furthermore, while preliminary dual-luciferase reporter assays have proven useful, clearly, increasingly thorough *in vivo* and *in vitro* experiments and approaches are needed in order to confirm the biological function of miRSNPs and their potential roles in PSD.

We analyzed the topological properties of PMSN and identified relatively novel potential significant roles of hsa04010 (MAPK signaling pathway) and for three high-risk genes (IGF1R, BNDF, and NTRK2). Further, we determined measures of associations of miRSNPs in cases of PSD. By searching for mechanisms underlying the dynamics of miRSNPs in genes identified as corresponding to high PSD risk, we were able to identify the three most potentially influential mechanisms related to the effects from the miRSNP → miRNA → risk gene → pathway. These three mechanisms were as follows: (i) rs28457673 (miR-15/16/195/424/497 family) → IGF1R → hsa04010 (MAPK signaling pathway)/hsa04151 (signaling pathway related to PI3K-Akt)/hsa01521 (resistance related to EGFR tyrosine kinase inhibitors), (ii) rs531564 (miR-124) → BDNF → hsa01521 (resistance related to EGFR tyrosine kinase inhibitors), and (iii) rs72739915 → (miR-20b/93) → NTRK2 → hsa04010 (MAPK signaling pathway)/hsa04151 (PI3K-Akt signaling pathway). Especially in relation to the rs28457673 (miR-15/16/195/424/497 family) → IGF1R → hsa04010 (MAPK signaling pathway), rs28457673 is located in the 3′-UTR of the IGF1R gene, which could affect the binding ability of IGF1R mRNA with miR-15/16/195/424/497 family. The activated IGF1R has been implicated in causing the activation of the Ras-MAPK pathway, thus inducing the inhibition of apoptosis and increasing protein synthesis. Previous research has indicated that abnormal IGF1R-mediated signaling in the brain may cause neurological sequelae. Findings also indicated that the inhibition of IGF1R in experimental models led to the partial improvement of neurogenesis and supported that there could be a potentially reversible nature of hippocampal-related changes (Andersson et al., 2018). These information strengthen our understanding of the potential mechanisms and functional roles that miRSNPs play in PSD pathogenesis. However, a lack of concrete and definitive experimental evidence for many of the steps we completed in our research means that the findings should be interpreted with some measures of caution. Regardless, we are certain that our novel approach has helped to demonstrate the value of multi-tiered studies and which has used the availability of richly populated and perhaps otherwise underutilized resources already existent in databases.

## Conclusions

In summary, we developed a catalog of risk based upon genes indicative of a high risk of PSD. We obtained information for PSD risk-related pathways and identified functionally oriented miRNAs and miRSNPs that appeared to have played important roles in the regulation of PSD risk-related pathways. Further, we built the PMSN and helped to elucidate the significant roles that the hsa04010 (MAPK signaling pathway), three high-risk genes, and related miRSNPs may play in PSD by way of examining the potential molecular mechanisms of miRSNPs that could have affected miRNA–mRNA interaction in PSD pathogenesis. The important next steps should include experiments both *in vitro* and *in vivo* in order to further confirm the possible roles of miRNAs and miRSNPs and how they may influence the dynamics and mechanisms underlying the onset, progression, and pathogenesis of PSD.

## Data Availability Statement

The original contributions presented in the study are included in the article/Supplementary Material, further inquiries can be directed to the corresponding authors.

## Author Contributions

S-hQ and S-wN designed the overall study. G-sX, A-yZ, and T-yZ collected and analyzed the data. M-xZ and G-bF wrote the original draft preparation. YL and Z-cW wrote, reviewed, and edited the manuscript. All authors have read and agreed to the published version of the manuscript.

## Conflict of Interest

The authors declare that the research was conducted in the absence of any commercial or financial relationships that could be construed as a potential conflict of interest.
