# Supplementary material for: Network and Pathway-Based Integrated Analysis Identified a Novel “rs28457673–miR-15/16/195/424/497 Family–IGF1R–MAPK Signaling Pathway” Axis Associated With Post-stroke Depression
Source: Front Cell Dev Biol. 2021 Jan 26;8:622424. doi: 10.3389/fcell.2020.622424 (PMC7870784; doi:10.3389/fcell.2020.622424)
Supplement: Supplementary file 1 [file Table_1.docx]

**Supplementary table S1. The catalog of post-stroke depression risk genes.**

| gene | Gene ID | Description | results | Expression | Detection method | Samples | Year | PMID |
| --- | --- | --- | --- | --- | --- | --- | --- | --- |
| HTR3D | 200909 | This gene encodes subunit D of the type 3 receptor for 5-hydroxytryptamine (serotonin), a biogenic hormone that functions as a neurotransmitter, a mitogen and a hormone. This hormone has been linked to neuropsychiatric disorders, including anxiety, depression, and migraine. Serotonin receptors causes fast and depolarizing responses in neurons following activation. | rs55674402, p=0.002512, odds ratio (OR)=0.7431 | Negative associations | Sequencing/ABI PRISM SNaPshot Multiplex Kit | discovery stage (121 PSD/131 stroke) ;replication stage (200 PSD/218 stroke | 2019 | 31121388([Fuying et al., 2019](#_ENREF_3)) |
| NEUROG3 | 50674 | The protein encoded by this gene is a basic helix-loop-helix (bHLH) transcription factor involved in neurogenesis. Defects in this gene are a cause of congenital malabsorptive diarrhea 4 (DIAR4) | rs144643855, p=0.00325, OR=0.6523 | Negative associations | Sequencing/ABI PRISM SNaPshot Multiplex Kit | discovery stage (121 PSD/131 stroke); replication stage (200 PSD/218 stroke | 2019 | 31121388([Fuying et al., 2019](#_ENREF_3)) |
| APOA4 | 337 | APOA4 (Apolipoprotein A4) is a Protein Coding gene. Diseases associated with APOA4 include Carotenemia and Demyelinating Polyneuropathy. Among its related pathways are Statin Pathway and Metabolism of fat-soluble vitamins. | ApoA-IV expression was significantly upregulated  in PSD as compared to stroke subjects | Upregulated  expression | iTRAQ coupled to a LC-MS/M or WB | 20 PSD/20 stroke | 2014 | 24853294([Zhan et al., 2014](#_ENREF_28)) |
| APOC2 | 344 | This gene encodes a lipid-binding protein belonging to the apolipoprotein gene family. The protein is secreted in plasma where it is a component of very low density lipoprotein. This protein activates the enzyme lipoprotein lipase, which hydrolyzes triglycerides and thus provides free fatty acids for cells. Mutations in this gene cause hyperlipoproteinemia type IB, characterized by hypertriglyceridemia, xanthomas, and increased risk of pancreatitis and early atherosclerosis. | ApoC-II expression were significantly downregulated in PSD relative to stroke subjects | Downregulated  expression | iTRAQ coupled to a LC-MS/M or WB | 20 PSD/20 stroke | 2014 | 24853294([Zhan et al., 2014](#_ENREF_28)) |
| GSN | 2934 | The protein encoded by this gene binds to the "plus" ends of actin monomers and filaments to prevent monomer exchange. The encoded calcium-regulated protein functions in both assembly and disassembly of actin filaments. Defects in this gene are a cause of familial amyloidosis Finnish type (FAF). | Gelsolin expression was  significantly dysregulated  in PSD relative to stroke subjects | Upregulated  expression | iTRAQ coupled to a LC-MS/M or WB | 20 PSD/20 stroke | 2014 | 24853294([Zhan et al., 2014](#_ENREF_28)) |
| HP | 3240 | This gene encodes a preproprotein, which is processed to yield both alpha and beta chains, which subsequently combine as a tetramer to produce haptoglobin. Mutations in this gene and/or its regulatory regions cause ahaptoglobinemia or hypohaptoglobinemia. This gene has also been linked to diabetic nephropathy, the incidence of coronary artery disease in type 1 diabetes, Crohn's disease, inflammatory disease behavior, primary sclerosing cholangitis, susceptibility to idiopathic Parkinson's disease, and a reduced incidence of Plasmodium falciparum malaria | Haptoglobin expression was  significantly dysregulated  in PSD relative to stroke subjects | Downregulated  expression | iTRAQ coupled to a LC-MS/MS or WB | 20 PSD/20 stroke | 2014 | 24853294([Zhan et al., 2014](#_ENREF_28)) |
| CFH | 3075 | This gene is a member of the Regulator of Complement Activation (RCA) gene cluster and encodes a protein with twenty short consensus repeat (SCR) domains. This protein is secreted into the bloodstream and has an essential role in the regulation of complement activation, restricting this innate defense mechanism to microbial infections. Mutations in this gene have been associated with hemolytic-uremic syndrome (HUS) and chronic hypocomplementemic nephropathy | Complement factor H expression were significantly downregulated in both PSD relative to stroke subjects(P<0.05) | Downregulated | iTRAQ coupled to a LC-MS/MS | 15 PSD/ 15 stroke | 2014 | 24853294([Zhan et al., 2014](#_ENREF_28)) |
| SERPINA3 | 12 | The protein encoded by this gene is a plasma protease inhibitor and member of the serine protease inhibitor class. Polymorphisms in this protein appear to be tissue specific and influence protease targeting. Variations in this protein's sequence have been implicated in Alzheimer's disease, and deficiency of this protein has been associated with liver disease. Mutations have been identified in patients with Parkinson disease and chronic obstructive pulmonary disease | Alpha-1-antichymotrypsin  expression were significantly downregulated in both PSD relative to stroke subjects(P<0.05) | Downregulated | iTRAQ coupled to a LC-MS/MS | 15 PSD/ 15 stroke | 2014 | 24853294([Zhan et al., 2014](#_ENREF_28)) |
| FGA | 2243 | This gene encodes the alpha subunit of the coagulation factor fibrinogen, which is a component of the blood clot. Following vascular injury, the encoded preproprotein is proteolytically processed by thrombin during the conversion of fibrinogen to fibrin. Mutations in this gene lead to several disorders, including dysfibrinogenemia, hypofibrinogenemia, afibrinogenemia and renal amyloidosis. | Fibrinogen alpha chain expression were significantly downregulated in both PSD relative to stroke subjects(P<0.05) | Downregulated | iTRAQ coupled to a LC-MS/MS | 15 PSD/ 15 stroke | 2014 | 24853294([Zhan et al., 2014](#_ENREF_28)) |
| APOA1 | 335 | This gene encodes apolipoprotein A-I, which is the major protein component of high density lipoprotein (HDL) in plasma. The encoded preproprotein is proteolytically processed to generate the mature protein, which promotes cholesterol efflux from tissues to the liver for excretion, and is a cofactor for lecithin cholesterolacyltransferase (LCAT), an enzyme responsible for the formation of most plasma cholesteryl esters. Defects in this gene are associated with HDL deficiencies, including Tangier disease, and with systemic non-neuropathic amyloidosis | Apolipoprotein A-I expression were significantly downregulated in both PSD relative to stroke subjects(P<0.05) | Downregulated | iTRAQ coupled with LC-MS/MS | 15 PSD/ 15 stroke | 2014 | 24853294([Zhan et al., 2014](#_ENREF_28)) |
| IGHM | 3507 | The IGHM gene encodes the C region of the mu heavy chain, which defines the IgM isotype. Naive B cells express the transmembrane forms of IgM and IgD (see IGHD; MIM 1471770) on their surface. During an antibody response, activated B cells can switch to the expression of individual downstream heavy chain C region genes by a process of somatic recombination known as isotype switching. In addition, secreted Ig forms that act as antibodies can be produced by alternative RNA processing of the heavy chain C region sequences | Ig mu chain C region expression were significantly downregulated in both PSD relative to stroke subjects(P<0.05) | Downregulated | iTRAQ coupled with LC-MS/MS | 15 PSD/ 15 stroke | 2014 | 24853294([Zhan et al., 2014](#_ENREF_28)) |
| SAA1 | 6288 | This gene encodes a member of the serum amyloid A family of apolipoproteins. This protein is a major acute phase protein that is highly expressed in response to inflammation and tissue injury. This protein also plays an important role in HDL metabolism and cholesterol homeostasis. High levels of this protein are associated with chronic inflammatory diseases including atherosclerosis, rheumatoid arthritis, Alzheimer's disease and Crohn's disease. This protein may also be a potential biomarker for certain tumors. | Serum amyloid A protein expression were significantly downregulated in both PSD relative to stroke subjects(P<0.05) | Downregulated | iTRAQ coupled with LC-MS/MS | 15 PSD/ 15 stroke | 2014 | 24853294([Zhan et al., 2014](#_ENREF_28)) |
| CFB | 629 | This gene encodes complement factor B, which circulates in the blood as a single chain polypeptide. Upon activation of the alternative pathway, it is cleaved by complement factor D yielding the noncatalytic chain Ba and the catalytic subunit Bb. Bb is involved in the proliferation of preactivated B lymphocytes, while Ba inhibits their proliferation. This cluster includes several genes involved in regulation of the immune reaction. Polymorphisms in this gene are associated with a reduced risk of age-related macular degeneration. | Complement factor B expression were significantly downregulated in both PSD relative to stroke subjects(P<0.05) | Downregulated | iTRAQ coupled with LC-MS/MS | 15 PSD/ 15 stroke | 2014 | 24853294([Zhan et al., 2014](#_ENREF_28)) |
| LRG1 | 116844 | The leucine-rich repeat (LRR) family of proteins, including LRG1, have been shown to be involved in protein-protein interaction, signal transduction, and cell adhesion and development. LRG1 is expressed during granulocyte differentiation | LRG expression were significantly downregulated in both PSD relative to stroke subjects | Downregulated  expression | iTRAQ coupled with LC-MS/M or WB | 20 PSD/20 stroke | 2014 | 24853294([Zhan et al., 2014](#_ENREF_28)) |
| CRP | 1401 | The protein encoded by this gene belongs to the pentaxin family. It is involved in several host defense related functions based on its ability to recognize foreign pathogens and damaged cells of the host and to initiate their elimination by interacting with humoral and cellular effector systems in the blood. Consequently, the level of this protein in plasma increases greatly during acute phase response to tissue injury, infection, or other inflammatory stimuli. | CRP expression were significantly downregulated in both PSD relative to stroke subjects | Downregulated | LC-MS/MS or WB | 20 PSD/20 stroke | 2014 | 24853294([Zhan et al., 2014](#_ENREF_28)) |
|  |  |  | Our results showed significantly higher Hs-CRP levels (1.54[IQR, 0.79–2.27]mg/dL vs. 0.43[IQR, 0.31–1.27] mg/dL, P<0.0001) at admission than patients without major depression. | Upregulated  expression | enzyme cycling method | 69 PSD/157 stroke | 2015 | 26042821([Yang et al., 2016](#_ENREF_25)) |
| IL-10 | 3586 | The protein encoded by this gene is a cytokine produced primarily by monocytes and to a lesser extent by lymphocytes. It down-regulates the expression of Th1 cytokines, MHC class II Ags, and costimulatory molecules on macrophages. It also enhances B cell survival, proliferation, and antibody production. This cytokine can block NF-kappa B activity, and regulate of the JAK-STAT signaling pathway. Mutations in this gene are associated with an increased susceptibility to HIV-1 infection and rheumatoid arthritis. | The major depression group had signifi cantly higher frequencies IL-10 – 1082 A ( χ 2 = 5.43, P = 0.018) alleles compared to those with no depression. | Positive association | PCR | 77 PSD/199 stroke | 2012 | 21793642([Kim et al., 2012b](#_ENREF_9)) |
| IL-4 | 3565 | Interleukin 4----The protein encoded by this gene is a pleiotropic cytokine produced by activated T cells. | The major depression group had significantly higher frequencies IL-4 +33 C (χ2=7.40, P=0.007) alleles compared to those with no depression. odds ratios, 8.18 (95%CI2.3–28.3; P= 0.001)]. | Positive association | PCR | 77 PSD/199 stroke | 2012 | 21793642([Kim et al., 2012b](#_ENREF_9)) |
| TNF | 7124 | The gene encodes a multifunctional proinflammatory cytokine that belongs to the tumor necrosis factor (TNF) superfamily. This cytokine is involved in the regulation of a wide spectrum of biological processes including cell proliferation, differentiation, apoptosis, lipid metabolism, and coagulation. Knockout studies in mice also suggested the neuroprotective function of this cytokine. | The major depression group had significantly higher frequencies TNF- α – 308 A (χ2 =5.28,P=0.022) alleles compared to those with no depression. | Positive association | PCR | 77 PSD/199 stroke | 2012 | 21793642([Kim et al., 2012b](#_ENREF_9)) |
| ApoE | 348 | Chylomicron remnants and very low density lipoprotein (VLDL) remnants are rapidly removed from the circulation by receptor-mediated endocytosis in the liver. Apolipoprotein E, a main apoprotein of the chylomicron, binds to a specific receptor on liver cells and peripheral cells. ApoE is essential for the normal catabolism of triglyceride-rich lipoprotein constituents. The APOE gene is mapped to chromosome 19 in a cluster with APOC1 and APOC2. | Expression of ApoE mRNA was significantly lower in  mononuclear blood cells in the PSD group (0.77 ± 0.24) than the control stroke group (0.86± 0.14) (p = 0.006 by t-test assuming unequal varianc)  The serum concentration of ApoE was significantly higher in the PSD group [(0.99 ±0.23) mg/dL] than the control stroke group [(0.85 ±0.28) mg/dL] （p = 0.048). | Downregulated | PCR  ELISA | 27PSD/ 34 stroke | 2013 | 23171142([Zhang et al., 2013](#_ENREF_31)) |
| TTR | 7276 | The encoded protein, transthyretin which transports thyroid hormones in the plasma and cerebrospinal fluid. It is also involved in the transport of retinol (vitamin A) in the plasma. The protein may also be involved in other intracellular processes including proteolysis, nerve regeneration, autophagy and glucose homeostasis. Mutations in this gene are associated with amyloid deposition, predominantly affecting peripheral nerves or the heart. | Serum prealbumin levels of patients PSD and non-PSD at admission were 24.077±6.84and28.437±7.65mg/dL, respectively, p<0.001.  In the logistic regression analysis, prealbumin  was independently associated with PSD (OR:0.942,95%CI:0.900–0.986, p=0.010) | Upregulated | immunoturbidimetric | 93 PSD/214 stroke | 2016 | 27693925([Wang et al., 2016](#_ENREF_21)) |
| NTRK2 | 4915 | This gene encodes a member of the neurotrophic tyrosine receptor kinase (NTRK) family. This kinase is a membrane-bound receptor that, upon neurotrophin binding, phosphorylates itself and members of the MAPK pathway. Signalling through this kinase leads to cell differentiation. Mutations in this gene have been associated with obesity and mood disorders. | The SNP rs1778929 was significantly more associated with incident PSD in participants with the TT genotype than in those with CC (OR 0.482, 95% CI: 0.313–0.744). In terms of rs1187323, stroke was significantly more associated with incident depression in participant with the AC genotype than in those with AA (OR 0.500, 95% CI: 0.368–0.680). | significantly associations | high-resolution melt (HRM) | 312 PSD/472 stroke | 2015 | 26641254([Zhiming Zhou, 2015](#_ENREF_32)) |
|  |  |  | For the TrkB gene, the frequencies of the haplotypes AT and AAT  were significantly lower in the PSD than the nonPSD groups (P=0.0251, OR = 0.622,95%CI=0.3845–0.9571 and P = 0.0306, OR =0.627, 95%CI=0.3852–0.9534, respectively). | Negative associations | sequencing | 122 PSD patients /  132 non PSD | 2017 | 29028593([Liang et al., 2018](#_ENREF_13)) |
| SLC6A4 | 6532 | This gene encodes an integral membrane protein that transports the neurotransmitter serotonin from synaptic spaces into presynaptic neurons. This protein is a target of psychomotor stimulants, such as amphetamines and cocaine, and is a member of the sodium:neurotransmitter symporter family. A repeat length polymorphism in the promoter of this gene has been shown to affect the rate of serotonin uptake. | Subjects with the 5-HTTLPR s/s genotype had three-fold higher odds for PSD compared to l/l or l/×l genotype carriers (OR 3.1, 95%CI 1.2–8.3). | significantly associations | PCR | 75PSD/75 stroke | 2008 | 18981341([Kohen et al., 2008](#_ENREF_11)) |
| STIN2-VNTR | 110806307 | This region can bind to transcription factors including CTCF and YB-1, and it may also co-operate with the 5-HTTLPR regulatory element, which is another VNTR polymorphic region located in the SLC6A4 promoter. Polymorphisms in this STin2 region may be associated with a variety of mood, behavioral and stress-related psychopathologies, possibly in a population-dependent fashion, in combination with other polymorphisms (such as the 5-HTTLPR), and/or related to epigenetic processes. | ubjects with the STin2 9/12 or 12/12 genotype had four-fold higher odds for PSD compared to STin2 10/10 genotype carriers (OR 4.1  95%CI 1.2–13.6). | significantly associations | PCR | 75PSD/75 stroke | 2008 | 18981341([Kohen et al., 2008](#_ENREF_11)) |
| IL1B | 3553 | The protein encoded by this gene is a member of the interleukin 1 cytokine family. This cytokine is produced by activated macrophages as a proprotein, which is proteolytically processed to its active form by caspase 1 (CASP1/ICE). This cytokine is an important mediator of the inflammatory response, and is involved in a variety of cellular activities, including cell proliferation, differentiation, and apoptosis. This gene and eight other interleukin 1 family genes form a cytokine gene cluster on chromosome 2. | Higher IL-1β (3.4±1.9pg/ml) concentrations were significantly associate d with PSD after 2 weeks, （2.6±1.2 pg/ml) | Upregulated  expression | solid-phase sandwich enzymelinked  immunosorbent assay kit ( | 80PSD/142 stroke | 2017 | 28844626([Kim et al., 2017](#_ENREF_7)) |
| ABCB1 | 5243 | The membrane-associated protein encoded by this gene is a member of the superfamily of ATP-binding cassette (ABC) transporters. ABC proteins transport various molecules across extra- and intra-cellular membranes. This protein is a member of the MDR/TAP subfamily. Members of the MDR/TAP subfamily are involved in multidrug resistance. The protein encoded by this gene is an ATP-dependent drug efflux pump for xenobiotic compounds with broad substrate specificity. It is responsible for decreased drug accumulation in multidrug-resistant cells and often mediates the development of resistance to anticancer drugs. This protein also functions as a transporter in the blood-brain barrier. Mutations in this gene are associated with colchicine resistance and Inflammatory bowel disease | Based on the CC genotype, the relative risk of homozygous mutant TT was 3.341 (x2 = 7.869; p = 0.005; OR= 3.341), and the T allele frequency in the PSD group was 49.4% higher than that in the NPSD group.  The locus gene frequency was 34.8%, and the relative risk of allele T relative to allele C was 1.830 (x2 = 8.381; p = 0.004; OR= 1.830). | significantly associations | fluorescence in situ  hybridization and chromosome karyotype analysis | 82PSD/115 stroke | 2019 | 31335167([Ding et al., 2019](#_ENREF_2)) |
| IL-6 | 3569 | This gene encodes a cytokine that functions in inflammation and the maturation of B cells. The protein is primarily produced at sites of acute and chronic inflammation, where it is secreted into the serum and induces a transcriptional inflammatory response through interleukin 6 receptor, alpha. The functioning of this gene is implicated in a wide variety of inflammation-associated disease states, including suspectibility to diabetes mellitus and systemic juvenile rheumatoid arthritis | The results showed that the ratio in PSD patients was significantly elevated in IL-6 (32.84±71.51 pg/ml) than non-PSD patients(1.47±4.28 pg/ml, P<.001) | Upregulated  expression | enzymelinked immunosorbent assay kits | 12PSD/92stroke | 2012 | 22055333([Su et al., 2012](#_ENREF_18)) |
|  |  |  | Higher IL-6 levels were independently associated  with depressive disorders within 2 weeks (23.2±18.0 vs 16.1±7.6,P=0.001) and at 1 year after stroke (23.1±19.5 vs16.8±8.3, P=0.025) | Upregulated  expression | ELISA | at baseline: 80 PSD/206 stroke  follow-up: 53 PSD/169 stroke | 2016 | 27428088([Kang et al., 2016](#_ENREF_6)) |
| IFNG | 3458 | This gene encodes a member of the type II interferon family. The protein encoded is a soluble cytokine with antiviral, immunoregulatory and anti-tumor properties and is a potent activator of macrophages. | PSD patients was significantly elevated in IL-6 (8.16±11.56 pg/ml) than non-PSD patients(0.21±1.28 pg/ml, P<.001) | Upregulated expression | enzymelinked immunosorbent assay kits | 12PSD/92 stroke | 2012 | 22055333([Su et al., 2012](#_ENREF_18)) |
| FNDC5 | 252995 | This gene encodes a secreted protein that is released from muscle cells during exercise. The encoded protein may participate in the development of brown fat. Translation of the precursor protein initiates at a non-AUG start codon at a position that is conserved as an AUG start codon in other organisms. | In the patients with depression, serum irisin levels were lower compared with those in patients without depression (P < 0.001). In a multivariate model using the first (Q1) quartile of irisin vs. Q2-4 together with the clinical variables, the marker displayed predictive information and increased risk of PSD by 75% (odds ratio [OR] for Q1, 1.75 [95% [CI], 1.15-2.65]). | Negative associations | ELISA | 370 PSD/835 stroke | 2018 | 29720216([Tu et al., 2018](#_ENREF_20)) |
| BDNF | 627 | This gene encodes a member of the nerve growth factor family of proteins. Alternative splicing results in multiple transcript variants, at least one of which encodes a preproprotein that is proteolytically processed to generate the mature protein. Binding of this protein to its cognate receptor promotes neuronal survival in the adult brain. Expression of this gene is reduced in Alzheimer's, Parkinson's, and Huntington's disease patients. This gene may play a role in the regulation of the stress response and in the biology of mood disorders | The result of meta analysis  demonstrated that serum levels of BDNF were significantly lower in patients with PSD compared with  patients without depression at the acute stage of stroke  (SMD = −1.43; 95% CI −2.56, −.31; P = .01) | Upregulated expression | ELISA | 171 PSD/328 stroke | 2018 | 29128330([Xu et al., 2018a](#_ENREF_22)) |
|  |  |  | For the BDNF gene, we determined that frequencies of the haplotypes GC and AG, which were composed of two SNPs, were significantly  higher in the PSD patients than the nonPSD patients (P = 0.0264, OR= 3.6635, 95%CI = 1.1642–11.5278 and P = 0.0138, OR = 6.7368, 95%CI = 1.4768–30.7317, respectively) | Positive association | sequencing | 122 PSD patients /132 stroke | 2017 | 29028593([Liang et al., 2018](#_ENREF_13)) |
| IL-18 | 3606 | interleukin 18 (interferon-gamma-inducing factor)--- The protein encoded by this gene is a proinflammatory cytokine that augments natural killer cell activity in spleen cells, and stimulates interferon gamma production in T-helper type I cells. | Higher IL-18 levels were independently associated  with depressive disorders within 2 weeks (313.6±104.2 VS 270.8±96.3,P=0.003) and at 1 year after stroke (305.8±111.4 VS275.0±96.3, P=0.026) | Upregulated expression | ELISA | at baseline: 80 PSD/206 stroke  follow-up: 53 PSD/169 stroke | 2016 | 27428088([Kang et al., 2016](#_ENREF_6)) |
| SERPINE1 | 5054 | This gene encodes a member of the serine proteinase inhibitor (serpin) superfamily. This member is the principal inhibitor of tissue plasminogen activator (tPA) and urokinase (uPA), and hence is an inhibitor of fibrinolysis. Defects in this gene are the cause of plasminogen activator inhibitor-1 deficiency (PAI-1 deficiency), and high concentrations of the gene product are associated with thrombophilia. | For the PAI-1 gene, the  rs72578597 polymorphism was significantly different between the PSD patients and the nonPSD patients (P = 0.025, using the Chi-square test) | Positive association | sequencing | 122 PSD patients /  132 stroke | 2017 | 29028593([Liang et al., 2018](#_ENREF_13)) |
| LEP | 3952 | This gene encodes a protein that is secreted by white adipocytes into the circulation and plays a major role in the regulation of energy homeostasis. Circulating leptin binds to the leptin receptor in the brain, which activates downstream signaling pathways that inhibit feeding and promote energy expenditure. This protein also has several endocrine functions, and is involved in the regulation of immune and inflammatory responses, hematopoiesis, angiogenesis, reproduction, bone formation and wound healing. Mutations in this gene and its regulatory regions cause severe obesity and morbid obesity with hypogonadism in human patients | Patients with major depression showed higher serum leptin levels at discharge [43.4 (23.4-60.2) v. 6.4 (3.7-16.8) ng/ml, p<0.001] and at 1 month after stroke [46.2 (34.0-117.7) v. 6.4 (3.4-12.2) ng/ml, p<0.001).  Serum levels of leptin >20.7 ng/ml were independently associated with post-stroke depression [OR 16.4, 95% (CI) 5.2-51.5, p<0.0001]. | Up regulated expression | ELISA | 23 PSD patients /  81 stroke | 2009 | 19356259([Jimenez et al., 2009](#_ENREF_5)) |
| INS | 3630 | This gene encodes insulin, a peptide hormone that plays a vital role in the regulation of carbohydrate and lipid metabolism. After removal of the precursor signal peptide, proinsulin is post-translationally cleaved into three peptides: the B chain and A chain peptides, which are covalently linked via two disulfide bonds to form insulin, and C-peptide. Binding of insulin to the insulin receptor (INSR) stimulates glucose uptake | Patients with major depression showed higher Insulin levels at baseline compared with patients without depression (IQR) 6.62(4.41–9.93) vs 4.14(3.33–4.94) mU/Ll, p<0.001] | Up regulated expression | enzyme-linked  immunosorbent assay ( | 186 PSD patients /  512 stroke | 2018 | 29408157([Qiu et al., 2018](#_ENREF_17)) |
| TAC1 | 6863 | This gene encodes four products of the tachykinin peptide hormone family, substance P and neurokinin A, as well as the related peptides, neuropeptide K and neuropeptide gamma. These hormones are thought to function as neurotransmitters which interact with nerve receptors and smooth muscle cells. They are known to induce behavioral responses and function as vasodilators and secretagogues. Substance P is an antimicrobial peptide with antibacterial and antifungal properties. | the level of plasma SP in the PSD group (58.47 +/- 14.39) was higher than that of the PS group (36.98 +/- 9.49; P = 0.000) | Up regulated | radioimmunoassay | 46 PSD patients /45 stroke | 2009 | 19566760([Li et al., 2009](#_ENREF_12)) |
| GPT | 2875 | This gene encodes cytosolic alanine aminotransaminase 1 (ALT1); also known as glutamate-pyruvate transaminase 1. This enzyme plays a key role in the intermediary metabolism of glucose and amino acids. Serum activity levels of this enzyme are routinely used as a biomarker of liver injury caused by drug toxicity, infection, alcohol, and steatosis. | the level of glutamate-pyruvate transaminase (GPT) in the PSD group (18 (15—24)) was lower than that of the PS group (22 (16—28) U L^−1^); P = 0.000) | Down-regulated | automated auto-analyzer | 70 PSD patients /139 stroke | 2014 | 25001962([Cheng et al., 2014](#_ENREF_1)) |
| GOT1 | 2805 | Glutamic-oxaloacetic transaminase is a pyridoxal phosphate-dependent enzyme which exists in cytoplasmic and mitochondrial forms, GOT1 and GOT2, respectively. GOT plays a role in amino acid metabolism and the urea and tricarboxylic acid cycles. The two enzymes are homodimeric and show close homology. | the level of GOT in the PSD group (14 (11—20)) was lower than that of the PS group (21 (15—32) U L−1); P = 0.000) | Down-regulated | automated auto-analyzer | 70 PSD patients /139 stroke | 2014 | 25001962([Cheng et al., 2014](#_ENREF_1)) |
| IGF1 | 3479 | The protein encoded by this gene is similar to insulin in function and structure and is a member of a family of proteins involved in mediating growth and development. It is known to increase the numbers of lymphocytes in the thymus and spleen, and enhance their function via greater lymphocyte generation and/or survival. IGF-I targets some progenitor cells of the immune sys-tem, including promyeloid, pro-B, and pro-T cells, promotes passage through the cell cycle, impairs cellular death, and increases differentiation to a more mature phenotype. | The serum IGF‐1 levels were significantly lower in PSD patients as compared with that of patients without depression (81 ng/mL [IQR,  99‐114] vs 124 ng/mL [IQR, 102‐154]; P < 0.001; | Down-regulated | ELISA | 74 PSD patients /151 stroke | 2017 | 28833493([Zhang et al., 2018](#_ENREF_29)) |
| ADIPOQ | 9370 | This gene is expressed in adipose tissue exclusively. It encodes a protein with similarity to collagens X and VIII and complement factor C1q. The encoded protein circulates in the plasma and is involved with metabolic and hormonal processes. Mutations in this gene are associated with adiponectin deficiency. | Patients with PSD showed lower level of APN (3.5 [2.5-6.3] μg/mL versus 6.2 [3.5-8.0] μg/mL, P = .001) at admission. Univariate logistic regression analysis indicated that patients with APN level in the first tertile compared with the third tertile were more likely to have PSD (odds ratio, 3.550; 95% confidence interval, 1.732-7.276; P = .008). | Down-regulated | ELISA | 69 PSD patients /186 stroke | 2018 | 30391327([Yang et al., 2019](#_ENREF_24)) |
| HTR2A | 3356 | This gene encodes one of the receptors for serotonin, a neurotransmitter with many roles. Mutations in this gene are associated with susceptibility to schizophrenia and obsessive-compulsive disorder, and are also associated with response to the antidepressant citalopram in patients with major depressive disorder (MDD). MDD patients who also have a mutation in intron 2 of this gene show a significantly reduced response to citalopram as this antidepressant downregulates expression of this gene. | Cases with any depression had significantly higher 5-HTR2a 1438 A allele  compared to controls with no  depression p=0.045. | Positive association | PCR-based restriction fragment length polymorphism | 77 PSD patients /199 nonPSD patients | 2012 | 22014446([Kim et al., 2012a](#_ENREF_8)) |
| HTR2C | 3358 | This gene encodes a seven-transmembrane G-protein-coupled receptor. The encoded protein responds to signaling through the neurotransmitter serotonin. The mRNA of this gene is subject to multiple RNA editing events, where adenosine residues encoded by the genome are converted to inosines. RNA editing is predicted to alter the structure of the second intracellular loop, thereby generating alternate protein forms with decreased ability to interact with G proteins. Abnormalities in RNA editing of this gene have been detected in victims of suicide that suffer from depression | There were significant associations between the HTR2C gene and PSD status in the male patients.  the rs12837651 T allele (odds ratio = 4.020) and the rs2192371 G allele (odds ratio = 2.866) were found to be significantly associated with PSD in males | significantly associations | Sequenom MassArray platform | 61 PSD patients /162 stroke | 2013 | 23765961([Tang et al., 2013](#_ENREF_19)) |
| NGF | 4803 | This gene is a member of the NGF-beta family and encodes a secreted protein which homodimerizes and is incorporated into a larger complex. This protein has nerve growth stimulating activity and the complex is involved in the regulation of growth and the differentiation of sympathetic and certain sensory neurons. | NGF (OR 0.06; P = 0.022) were independent biochemical predictors of  PSD (P < 0.05) ( | significantly associations | enzyme-linked immunosorbent | 36PSD patients /47 stroke | 2017 | 29149884([Meng et al., 2017](#_ENREF_16)) |
| CAT | 847 | This gene encodes catalase, a key antioxidant enzyme in the bodies defense against oxidative stress. Catalase is a heme enzyme that is present in the peroxisome of nearly all aerobic cells. Catalase converts the reactive oxygen species hydrogen peroxide to water and oxygen and thereby mitigates the toxic effects of hydrogen peroxide. | Serum levels of CAT (P=0.025) increased in depressed patients at admission.  A positive correlation CAT levels in and the depressed patients (r=0.300, P=0.012). | Positive association | enzyme-linked immunosorbent assay | 70 PSD patients /171 nonPSD patients | 2018 | 29497302([Liu et al., 2018](#_ENREF_14)) |
| MIF | 4282 | This gene encodes a lymphokine involved in cell-mediated immunity, immunoregulation, and inflammation. It plays a role in the regulation of macrophage function in host defense through the suppression of anti-inflammatory effects of glucocorticoids. | plasma levels of MIF were higher compared with those in patients free-depression [27.3(IQR, 23.5-34.9) ng/ml vs. 20.9(IQR, 17.0-24.8) ng/ml; Z = 8.369, P < 0.001]. | significantly associations | Quantikine Human MIF Immunoassay | 95 PSD patients /238 stroke | 2018 | 29759141([Xu et al., 2018b](#_ENREF_23)) |
| TPH2 | 121278 | This gene encodes a member of the pterin-dependent aromatic acid hydroxylase family. The encoded protein catalyzes the first and rate limiting step in the biosynthesis of serotonin, an important hormone and neurotransmitter. Mutations in this gene may be associated with psychiatric diseases such as bipolar affective disorder and major depression | The TPH2 rs4641528 genotype frequencies differed significantly between patients with and without either PSD. In multiple logistic regression  analysis, PSD was related to the TPH2 rs4641528 C allele (95% CI: 1.039–5.631, p < .05), | significantly association | PCR | 69 PSD patients /314 stroke | 2018 | 29484259([Ko et al., 2018](#_ENREF_10)) |
| GDNF | 2668 | This gene encodes a secreted ligand of the TGF-beta (transforming growth factor-beta) superfamily of proteins. The recombinant form of this protein, a highly conserved neurotrophic factor, was shown to promote the survival and differentiation of dopaminergic neurons in culture, and was able to prevent apoptosis of motor neurons induced by axotomy. Mutations in this gene may be associated with Hirschsprung disease and Tourette syndrome | The spearman analysis showed that GDNF protein level negatively correlated with the value of HAMD-17 in PSD patients | Positive association | qRT-PCR  ELISA | 39 PSD patients /42 stroke | 2017 | 28819313([Zhang et al., 2017](#_ENREF_30)) |
| CREB1 | 1385 | This gene encodes a transcription factor that is a member of the leucine zipper family of DNA binding proteins. This protein binds as a homodimer to the cAMP-responsive element, an octameric palindrome. The protein is phosphorylated by several protein kinases, and induces transcription of genes in response to hormonal stimulation of the cAMP pathway | CREB1 rs139164200, with significant association with PSD(p=0.0001165,  OR=2.281) | significantly association | Sequencing | discovery stage 121 PSD/131 stroke | 2019 | 31121388([Fuying et al., 2019](#_ENREF_3)) |
| MTHFR | 4524 | The protein encoded by this gene catalyzes the conversion of 5,10-methylenetetrahydrofolate to 5-methyltetrahydrofolate.Genetic variation in this gene influences susceptibility to occlusive vascular disease, neural tube defects, colon cancer and acute leukemia, and mutations in this gene are associated with methylenetetrahydrofolate reductase | The 677T allele and 677C/T genotype of methylenetetrahydrofolate reductase gene 677C>T polymorphism, which were over-represented in depression patients and were respectively associated with 1.82-fold (p=0.001) and 3.65-fold (p<0.001) increased risk of post-stroke depression relative to the 677C allele and 677C/C genotype | significantly association |  | 106 PSD/175 stroke | 2018 | 30174331([Mei F, 2018](#_ENREF_15)) |
| NPY | 4852 | This gene encodes a neuropeptide that is widely expressed in the central nervous system and influences many physiological processes, including cortical excitability, stress response, food intake, circadian rhythms, and cardiovascular function. The neuropeptide inhibit adenylyl cyclase, activate mitogen-activated protein kinase (MAPK), regulate intracellular calcium levels, and activate potassium channels. A polymorphism in this gene resulting in a change of leucine 7 to proline in the signal peptide is associated with elevated cholesterol levels, higher alcohol consumption, and may be a risk factor for various metabolic and cardiovascular diseases. | Serum NPY concentration and mRNA expression were independent predictors of PSD with OR of 0.933  (95% CI,0.871–1.000; P = 0.050),1.452 (95%CI,1.081–1.951;P = 0.013), respectively | Protien UP-regulated/  mRNA-  down-regulate | ELISA  qPCR | 39 PSD/42 stroke | 2016 | 28082897([Yue et al., 2016](#_ENREF_27)) |
| IGF1R | 3480 | This receptor binds insulin-like growth factor with a high affinity. It has tyrosine kinase activity. The insulin-like growth factor I receptor plays a critical role in transformation events. It is highly overexpressed in most malignant tissues where it functions as an anti-apoptotic agent by enhancing cell survival. | Compared with non-PSD , IGF-1R protein in PSD increased（243.32± 146.69 vs 171.16 50.85,p<0.05 | UP-regulated | WB | 38 PSD/42 non-PSD | 2016 | 27527872([Yingying Yue, 2016](#_ENREF_26)) |
| S100B | 6285 | S100 proteins are localized in the cytoplasm and/or nucleus of a wide range of cells, and involved in the regulation of a number of cellular processes such as cell cycle progression and differentiation. his protein may function in Neurite extension, proliferation of melanoma cells, stimulation of Ca2+ fluxes, inhibition of PKC-mediated phosphorylation, astrocytosis and axonal proliferation, and inhibition of microtubule assembly | When patients were subdivided into two groups according to the GDS score (GDS≤8 and GDS>8), b S100B concentrations were higher in patients with GDS>8.  S100B was found to be independently associated with the geriatric depression  score (F=8.150, p=0.009), | significantly association | ELISA | 10 PSD/ 15 non-PSD |  | 22820433([Gonzalez-Garcia et al., 2012](#_ENREF_4)) |
| ENO2 | 2026 | This gene encodes one of the three enolase isoenzymes found in mammals. This isoenzyme, a homodimer, is found in mature neurons and cells of neuronal origin. A switch from alpha enolase to gamma enolase occurs in neural tissue during development in rats and primates. | When patients were subdivided into two groups according to the GDS score (GDS≤8 and GDS>8), NSE concentrations were higher in patients with GDS>8. | UP-regulated | ELISA | 9 PSD/ 13 non-PSD | 2012 | 22820433([Gonzalez-Garcia et al., 2012](#_ENREF_4)) |
| KDR | 3791 | This gene encodes one of the two receptors of the VEGF. This receptor, known as kinase insert domain receptor, is a type III receptor tyrosine kinase. It functions as the main mediator of VEGF-induced endothelial proliferation, survival, migration, tubular morphogenesis and sprouting. Mutations of this gene are implicated in infantile capillary hemangiomas. | Compared with normal controls (NC), there was decreased VEGFR2 content in PSD (P < 0.05, 95%  (CI), -672.21~-306.37), | Down-regulated | WB | 39 PSD/ 38 normol | 2016 | 27527872([Yingying Yue, 2016](#_ENREF_26)) |
| PIGF | 5281 | This gene encodes a protein involved in glycosylphosphatidylinositol (GPI)-anchor biosynthesis. The GPI-anchor, a glycolipid containing three mannose molecules in its core backbone, is found on many blood cells where it serves to anchor proteins to the cell surface. The encoded protein and another GPI synthesis protein, PIGO, function in the transfer of ethanolaminephosphate to the third mannose in GPI. | Compared with normal controls (NC), increased  PIGF protein in PSD (P < 0.05,  95%CI, 3.92~9.04) | UP-regulated | PCR | 38 PSD/ 37 normol | 2016 | 27527872([Yingying Yue, 2016](#_ENREF_26)) |
| VEGFA | 7422 | This gene is a member of the PDGF/VEGF growth factor family. It encodes a heparin-binding protein, which exists as a disulfide-linked homodimer. This growth factor induces proliferation and migration of vascular endothelial cells, and is essential for both physiological and pathological angiogenesis. Disruption of this gene in mice resulted in abnormal embryonic blood vessel formation. | Compared with normal controls (NC), PSD had  decreased VEGF in mRNA field by post hoc non parametric test ( P <0.05). | Down-regulated | PCR | 38 PSD/ 38 normol | 2016 | 27527872([Yingying Yue, 2016](#_ENREF_26)) |

Cheng, S.Y., Zhao, Y.D., Li, J., Chen, X.Y., Wang, R.D., and Zeng, J.W. (2014). Plasma levels of glutamate during stroke is associated with development of post-stroke depression. *Psychoneuroendocrinology* 47**,** 126-135. doi: 10.1016/j.psyneuen.2014.05.006.

Ding, X., Yang, Q., Su, L., Huang, X., and Zhou, Z. (2019). Correlation Between C3435T Locus of ABCB1 Gene and Poststroke Depression in China. *DNA and Cell Biology* 38(8)**,** 808-813. doi: 10.1089/dna.2018.4565.

Fuying, Z., Yingying, Y., Shining, Z., Kezhong, Z., Yanyan, S., Xuemei, Z., et al. (2019). Novel susceptibility genes were found in a targeted sequencing of stroke patients with or without depression in the Chinese Han population. *J Affect Disord* 255**,** 1-9. doi: 10.1016/j.jad.2019.05.023.

Gonzalez-Garcia, S., Gonzalez-Quevedo, A., Fernandez-Concepcion, O., Pena-Sanchez, M., Menendez-Sainz, C., Hernandez-Diaz, Z., et al. (2012). Short-term prognostic value of serum neuron specific enolase and S100B in acute stroke patients. *Clin Biochem* 45(16-17)**,** 1302-1307. doi: 10.1016/j.clinbiochem.2012.07.094.

Jimenez, I., Sobrino, T., Rodriguez-Yanez, M., Pouso, M., Cristobo, I., Sabucedo, M., et al. (2009). High serum levels of leptin are associated with post-stroke depression. *Psychol Med* 39(7)**,** 1201-1209. doi: 10.1017/S0033291709005637.

Kang, H.J., Bae, K.Y., Kim, S.W., Kim, J.T., Park, M.S., Cho, K.H., et al. (2016). Effects of interleukin-6, interleukin-18, and statin use, evaluated at acute stroke, on post-stroke depression during 1-year follow-up. *Psychoneuroendocrinology* 72**,** 156-160. doi: 10.1016/j.psyneuen.2016.07.001.

Kim, J.M., Kang, H.J., Kim, J.W., Bae, K.Y., Kim, S.W., Kim, J.T., et al. (2017). Associations of Tumor Necrosis Factor-alpha and Interleukin-1beta Levels and Polymorphisms with Post-Stroke Depression. *Am J Geriatr Psychiatry* 25(12)**,** 1300-1308. doi: 10.1016/j.jagp.2017.07.012.

Kim, J.M., Stewart, R., Bae, K.Y., Kim, S.W., Kang, H.J., Shin, I.S., et al. (2012a). Serotonergic and BDNF genes and risk of depression after stroke. *J Affect Disord* 136(3)**,** 833-840. doi: 10.1016/j.jad.2011.09.029.

Kim, J.M., Stewart, R., Kim, S.W., Shin, I.S., Kim, J.T., Park, M.S., et al. (2012b). Associations of cytokine gene polymorphisms with post-stroke depression. *World J Biol Psychiatry* 13(8)**,** 579-587. doi: 10.3109/15622975.2011.588247.

Ko, M., Choi-Kwon, S., Jun, S.E., Kim, J.H., Cho, K.H., Nah, H.W., et al. (2018). Poststroke emotional disturbances and a tryptophan hydroxylase 2 gene polymorphism. *Brain Behav* 8(2)**,** e00892. doi: 10.1002/brb3.892.

Kohen, R., Cain, K.C., Mitchell, P.H., Becker, K., Buzaitis, A., Millard, S.P., et al. (2008). Association of serotonin transporter gene polymorphisms with poststroke depression. *Arch Gen Psychiatry* 65(11)**,** 1296-1302. doi: 10.1001/archpsyc.65.11.1296.

Li, L., Gao, X., Zhao, J., Ji, X., Wei, H., and Luo, Y. (2009). Plasma and cerebrospinal fluid substance P in post-stroke patients with depression. *Psychiatry Clin Neurosci* 63(3)**,** 298-304. doi: 10.1111/j.1440-1819.2009.01936.x.

Liang, J., Yue, Y., Jiang, H., Geng, D., Wang, J., Lu, J., et al. (2018). Genetic variations in the p11/tPA/BDNF pathway are associated with post stroke depression. *J Affect Disord* 226**,** 313-325. doi: 10.1016/j.jad.2017.09.055.

Liu, Z., Cai, Y., and He, J. (2018). High serum levels of 8-OHdG are an independent predictor of post-stroke depression in Chinese stroke survivors. *Neuropsychiatr Dis Treat* 14**,** 587-596. doi: 10.2147/NDT.S155144.

Mei F, W.Y., Ding G, Pan F, Chen L, Wu J. (2018). Association of methylenetetrahydrofolate reductase gene 677C>Tpolymorphism with post-stroke depression risk and antidepressant treatment response in Han Chinese. *J Pak Med Assoc.* 68(7)**,** 4.

Meng, G., Ma, X., Li, L., Tan, Y., Liu, X., Liu, X., et al. (2017). Predictors of early-onset post-ischemic stroke depression: a cross-sectional study. *BMC Neurol* 17(1)**,** 199. doi: 10.1186/s12883-017-0980-5.

Qiu, H.C., Liu, H.Z., Li, X., Zeng, X., and Zhao, J.Z. (2018). Insulin resistance as estimated by homeostasis model assessment predicts incident post-stroke depression in Chinese subjects from ischemic stroke. *J Affect Disord* 231**,** 1-7. doi: 10.1016/j.jad.2018.01.023.

Su, J.A., Chou, S.Y., Tsai, C.S., and Hung, T.H. (2012). Cytokine changes in the pathophysiology of poststroke depression. *Gen Hosp Psychiatry* 34(1)**,** 35-39. doi: 10.1016/j.genhosppsych.2011.09.020.

Tang, W.K., Tang, N., Liao, C.D., Liang, H.J., Mok, V.C., Ungvari, G.S., et al. (2013). Serotonin receptor 2C gene polymorphism associated with post-stroke depression in Chinese patients. *Genet Mol Res* 12(2)**,** 1546-1553. doi: 10.4238/2013.May.13.8.

Tu, W.J., Qiu, H.C., Liu, Q., Li, X., Zhao, J.Z., and Zeng, X. (2018). Decreased level of irisin, a skeletal muscle cell-derived myokine, is associated with post-stroke depression in the ischemic stroke population. *J Neuroinflammation* 15(1)**,** 133. doi: 10.1186/s12974-018-1177-6.

Wang, L., Xu, H., Ren, W., Zhu, L., Chang, Y., Gu, Y., et al. (2016). Low serum prealbumin levels in post-stroke depression. *Psychiatry Res* 246**,** 149-153. doi: 10.1016/j.psychres.2016.09.021.

Xu, H.B., Xu, Y.H., He, Y., Xue, F., Wei, J., Zhang, H., et al. (2018a). Decreased Serum Brain-Derived Neurotrophic Factor May Indicate the Development of Poststroke Depression in Patients with Acute Ischemic Stroke: A Meta-Analysis. *J Stroke Cerebrovasc Dis* 27(3)**,** 709-715. doi: 10.1016/j.jstrokecerebrovasdis.2017.10.003.

Xu, T., Pu, S., Ni, Y., Gao, M., Li, X., and Zeng, X. (2018b). Elevated plasma macrophage migration inhibitor factor as a risk factor for the development of post-stroke depression in ischemic stroke. *J Neuroimmunol* 320**,** 58-63. doi: 10.1016/j.jneuroim.2018.04.003.

Yang, J., Du, G., Wang, J., Chen, J., Yang, C., Li, J., et al. (2019). Reduced Serum Adiponectin Level and Risk of Poststroke Depression in Patients with Ischemic Stroke. *J Stroke Cerebrovasc Dis* 28(2)**,** 305-310. doi: 10.1016/j.jstrokecerebrovasdis.2018.09.057.

Yang, R.R., Lu, B.C., Li, T., Du, Y.F., Wang, X., and Jia, Y.X. (2016). The relationship between high-sensitivity C-reactive protein at admission and post stroke depression: a 6-month follow-up study. *Int J Geriatr Psychiatry* 31(3)**,** 231-239. doi: 10.1002/gps.4315.

Yingying Yue, L., Shenghua Li,Haitang Jiang,Jun Wang, Rui Liu, Jianxin Lu, Yingying Yin, Deqin Geng, Yuqun Zhang, Aiqin Wu, Jinfeng and Yonggui Yuan (2016). Towards a multi protein and mRNA expression of biological predictive and distinguish model for post stroke depression. *Oncotarget* Vol. 7(No. 34)**,** 10.

Yue, Y., Jiang, H., Yin, Y., Zhang, Y., Liang, J., Li, S., et al. (2016). The Role of Neuropeptide Y mRNA Expression Level in Distinguishing Different Types of Depression. *Front Aging Neurosci* 8**,** 323. doi: 10.3389/fnagi.2016.00323.

Zhan, Y., Yang, Y.T., You, H.M., Cao, D., Liu, C.Y., Zhou, C.J., et al. (2014). Plasma-based proteomics reveals lipid metabolic and immunoregulatory dysregulation in post-stroke depression. *Eur Psychiatry* 29(5)**,** 307-315. doi: 10.1016/j.eurpsy.2014.03.004.

Zhang, W., Wang, W., and Kuang, L. (2018). The relation between insulin-like growth factor 1 levels and risk of depression in ischemic stroke. *Int J Geriatr Psychiatry* 33(2)**,** e228-e233. doi: 10.1002/gps.4774.

Zhang, Y., Jiang, H., Yue, Y., Yin, Y., Zhang, Y., Liang, J., et al. (2017). The protein and mRNA expression levels of glial cell line-derived neurotrophic factor in post stroke depression and major depressive disorder. *Sci Rep* 7(1)**,** 8674. doi: 10.1038/s41598-017-09000-y.

Zhang, Z., Mu, J., Li, J., Li, W., and Song, J. (2013). Aberrant apolipoprotein E expression and cognitive dysfunction in patients with poststroke depression. *Genet Test Mol Biomarkers* 17(1)**,** 47-51. doi: 10.1089/gtmb.2012.0253.

Zhiming Zhou, X.D., Qian Yang, Jia Hu, Xianjin Shang, Xianjun Huang, Liang Ge, Taofeng Zhou (2015). Association between Single-Nucleotide Polymorphisms of the Tyrosine Kinase Receptor B (TrkB) and Post-Stroke Depression in China. *PLoS ONE* 10(12)**,** e0144301. doi: 10.6084/m9.figshare.1608715

10.1371/journal.pone.0144301.
